# [Corrigendum] Effects of Platycodin D on apoptosis, migration, invasion and cell cycle arrest of gallbladder cancer cells

**DOI:** 10.3892/ol.2026.15780

**Published:** 2026-07-27

**Authors:** Xiaoyu Zhang, Tianyu Zhai, Zhenyu Hei, Di Zhou, Longyang Jin, Chao Han, Jiandong Wang

Oncol Lett 20: 311, 2020; DOI: 10.3892/ol.2020.12174

Subsequently to the publication of the above article, an interested reader drew to the authors’ attention that, on p. 6, the data panels showing the results of cellular migration assay experiments in Fig. 5A, and those showing the results of the invasion assay experiments in Fig. 5C, contained overlapping sections of data. Specifically, the ‘NOZ/Control’ and ‘NOZ/5 μM platcodin (PD)’ data panels in Fig. 5A, and the ‘GBC-SD/Control’, ‘GBC-SD/5 μM PD’ and ‘GBC-SD/10 μM PD’ data panels in Fig. 5C, contained overlapping sections of data, such that these panels were apparently derived from the same original sources where the results of differently performed experiments were intended to have been portrayed.

After re-examining their original data, the authors have realized that the data in question were inadvertently assembled incorrectly in Fig. 5A and C. The revised and corrected version of Fig. 5, now including the correct data for the ‘NOZ/5 μM PD’ data panel in Fig. 5A and data from one of the repeated sets of invasion assay experiments for the GBC-SD cell line in Fig. 5C, is shown on the next page. The results from the repeated experiments were very similar to those featured originally in the paper. The authors are grateful to the Editor of *Oncology Letters* for allowing them this opportunity to publish a Corrigendum, and all the authors agree with its publication. Furthermore, the authors apologize to the readership for any inconvenience caused.

## Figures and Tables

**Figure 5.**
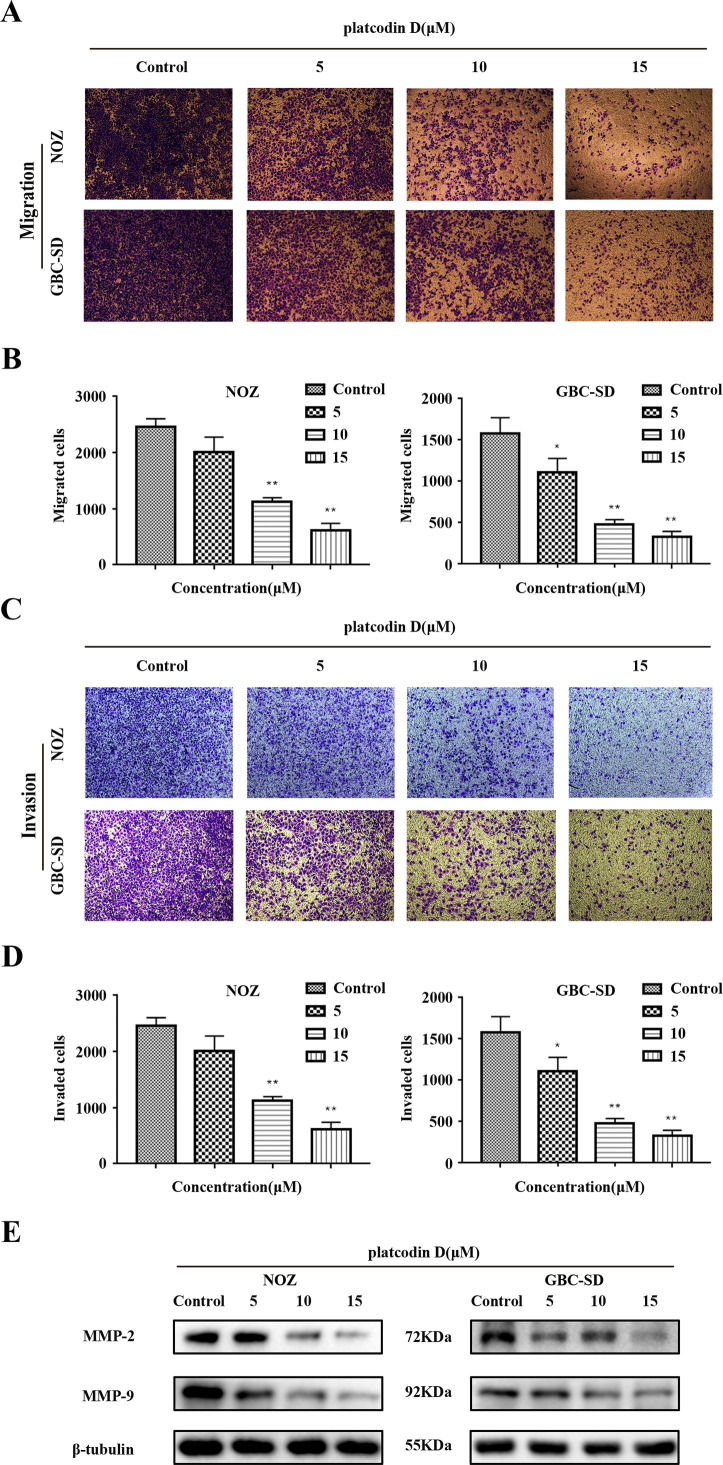
PD inhibits the migration and invasion of gallbladder cancer cells. (A) Migrative ability was determined via cell migration assays, and the migrated cells were stained and observed (magnification ×100). (B) Number of migrated cells was counted as shown. (C) Invasive ability was determined via cell invasion assays, and the invaded cells were stained and observed (magnification ×100). (D) Number of invading cells was counted as shown. (E) MMP-2 and MMP-9 expression was detected via western blotting. Each experiment was repeated three times independently. *P<0.05, **P<0.01 vs. control. PD, Platycodin D.

